# Acute Brugian filariasis in a German tourist after short-term travel to Sri Lanka, 2025

**DOI:** 10.2807/1560-7917.ES.2025.30.43.2500793

**Published:** 2025-10-30

**Authors:** Günther Slesak, Birgit Muntau, Dennis Tappe

**Affiliations:** 1 Tropenklinik Paul-Lechler-Hospital, Department of Tropical Medicine, Tübingen, Germany; 2 Bernhard Nocht Institute for Tropical Medicine, Hamburg, Germany

**Keywords:** acute filariasis, *Brugia malayi*, travel, Sri Lanka

## Abstract

A German traveller developed leg oedema with adenolymphangitis after a 3-week trip to Sri Lanka. Laboratory tests showed slight eosinophilia, positive filarial serology and motile microfilariae in day and night-time blood samples. PCR revealed 99% homology for *Brugia malayi*. Lymphatic filariasis has been eliminated in Sri Lanka, but zoonotic *B. malayi* has re-emerged. Physicians need to be alert and consider filariasis as potential differential diagnosis. Surveillance and vector control efforts should be sustained to prevent resurgence in Sri Lanka.

Lymphatic filariasis is a neglected tropical parasitic disease transmitted to humans through blood-feeding mosquitoes [1,2]. Together with several other countries, Sri Lanka has achieved the status of elimination of lymphatic filariasis as a public health problem [3,4]. Thus, similar to malaria, lymphatic filariasis has vanished as a differential diagnosis in returning travellers from Sri Lanka. Here we describe a case of acute lymphatic filariasis in a German tourist after a short-term trip to Sri Lanka and discuss implications for public and travellers’ health.

## Case report

A previously healthy middle-aged traveller presented with oedema of the left leg 4 months after a 3-week trip to south-western Sri Lanka in April 2025. The patient remembered repeated mosquito bites during the visit. Initially, some localised swelling at the inner proximal thigh had developed 2 months after return which slowly spread distally to the ankle. The patient had already received empiric antimicrobial therapy and venous thrombosis had been excluded twice, including by ultrasound.

**Figure 1:**
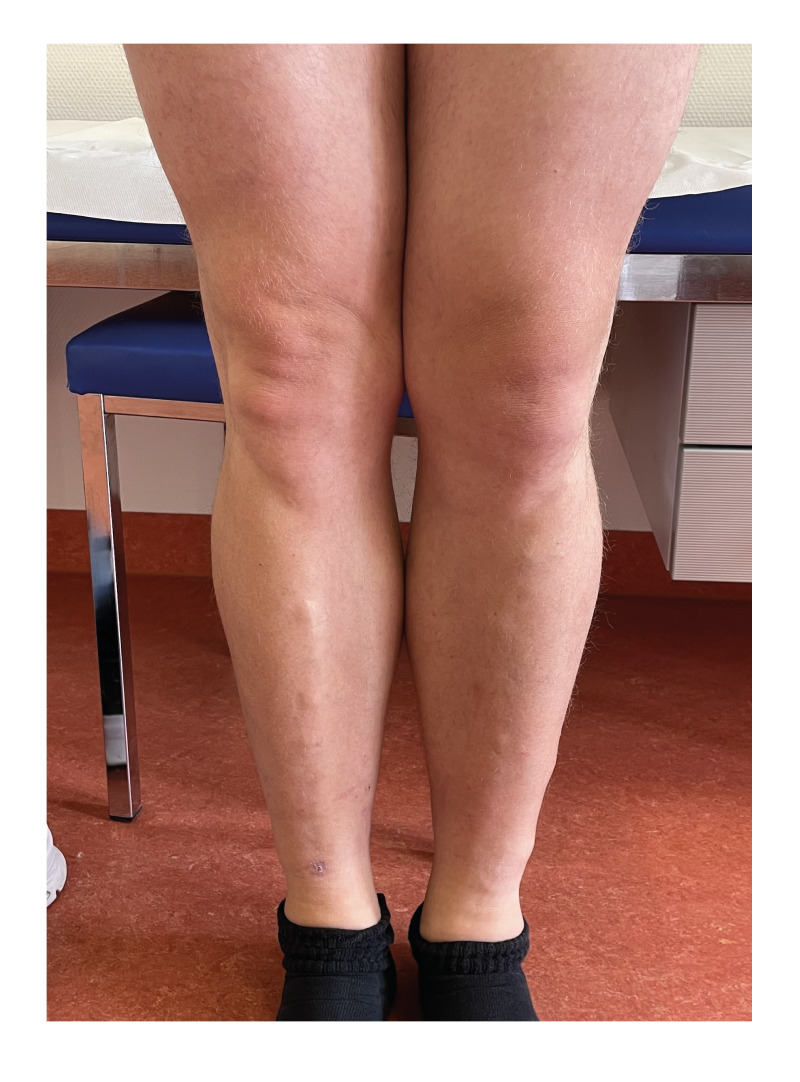
Diffuse swelling of the left leg of a patient with lymphatic filariasis after a visit to Sri Lanka, 2025

**Figure 2:**
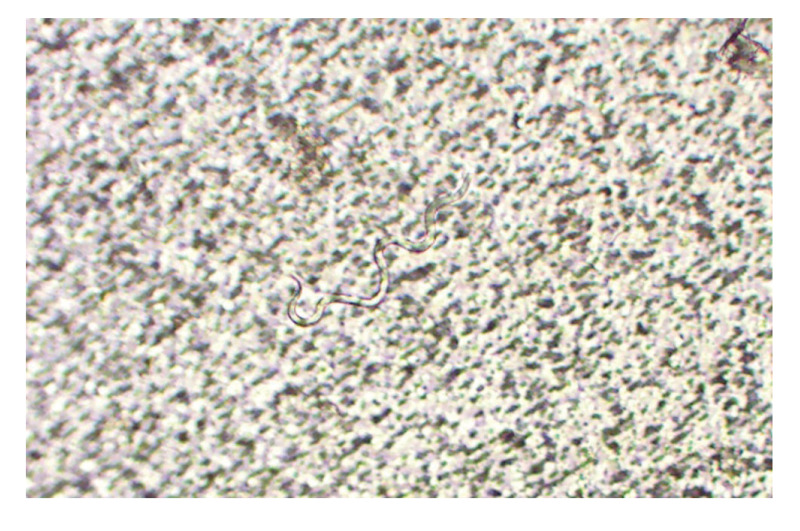
Motile microfilaria in unstained blood smear after filtration of a sample taken at daytime from a patient with lymphatic filariasis after a visit to Sri Lanka, 2025

On examination, the patient had enlarged tender femoral lymph nodes and diffuse swelling especially around the left knee and ancle (Figure 1). Blood tests showed slight eosinophilia of 500/µL (norm: < 450/µL) and antibodies against filarial antigens in a *Dirofilaria immitis* crude antigen in-house ELISA (118 arbitrary antibody units; norm: < 10 units, as determined by testing 24 samples of healthy blood donors). The test has a clinical sensitivity for filariasis of 85% and a specificity of 82%. Unstained blood smears before and after filtration from day and night-time samples revealed few motile microfilariae (Figure 2). In the Supplementary Video, the motile microfilariae are visible. Further microscopic identification by Giemsa stain was unsuccessful, but nematode-specific 12S rDNA PCR [5] was positive from a paper filter used for EDTA whole blood. Sequencing of the 407 bp amplicon revealed 99% homology with GenBank entries (NC_004298, and others; https://www.ncbi.nlm.nih.gov/genbank) for *Brugia malayi*. Ultrasonography showed enlarged femoral lymph nodes of up to 1.9 cm of size but no adult worms. The patient was started on doxycycline (200 mg/day for 5 weeks), and later albendazole (400 mg/day for 1 week) was added [6]. Follow-up at the end of the treatment revealed only very mild swelling of the ancle but normal blood tests except of one microfilaria in a filtered blood sample.

Magnification: 100 ×.

## Discussion

In 2016, Sri Lanka was declared to have eliminated lymphatic filariasis as a public health problem, after national programmes with annual rounds of mass drug administrations had been successfully conducted [4]. However, in contrast to the most prevalent pathogen in Asia, *Wuchereria bancrofti* [2], *B. malayi* is also causing zoonoses, with dogs, cats and monkeys as reservoir hosts of the parasite [7,8]. Vectors are mainly night-biting mosquitoes of various *Mansonia* and *Anopheles* species [9,10]. They are widely present in tropical and subtropical Asia, yet some (e.g. *Mansonia uniformis*) commonly occur from Western Africa to Australia [11,12]. A current study found evidence for a much wider range of potential vectors for *B. malayi* in Sri Lanka, including several *Culex* and *Aedes* species [13].

After elimination of *B. malayi* in Sri Lanka in the late 1960s [14], human Brugian filariasis has re-emerged in the early 2000s [8], especially in south-western wet areas [7]. In vertebrate hosts, microfilariae are observed in the peripheral blood vessels throughout the day, peaking at night (nocturnal sub-periodicity) and contrast to the nocturnal periodic former *B. malayi* strain which suggests a zoonotic origin [8,15] of a novel genetic variant [16]. Recent entomological studies in Gampaha, western Sri Lanka have shown that up to 21% of *Mansonia* mosquitoes carried *B. malayi* [10]. Moreover, *B. malayi* microfilarial rates of up to 69% were detected in dogs in rural areas of western Sri Lanka [17].

Sri Lanka has become a popular tourist destination in Asia and has recorded a further increase in international travellers by 16.2% so far in 2025, the majority coming from Europe [18]. Our case highlights the risk of filariasis not solely for residents of Sri Lanka but also travellers to Sri Lanka. Physicians should be aware of this condition and its various differential diagnoses as highlighted by the patient’s history. Key for early case detection and treatment of acute filarial lymphangitis is the combination of eosinophilia and clinical signs with adenolymphangitis and oedema spreading distally weeks to months after exposure. Diagnostic workout needs to consider the latency (parasite prepatence) of 3–7 months until microfilariae become microscopically visible in blood samples. Combination treatment can achieve higher filarial clearance rates than single drug antifilarial therapy [6,19] and has successfully been used in elimination programmes [2,4].

## Conclusion

Further efforts for surveillance and vector control in a One Health approach are imperative to impede resurgence of human filariasis in Sri Lanka and prevent travel-related spread of Brugian filariasis to other areas of the world where zoonotic hosts and potential vectors exist.

## Data Availability

Data on PCR sequences are presented in Supplement2.

## Supplementary Data

10514000 Supplementary Material 1

## Supplementary Data

87000 Supplementary Material 2
