# Supplementary material for: Acute Brugian filariasis in a German tourist after short-term travel to Sri Lanka, 2025
Source: Euro Surveill. 2025 Oct 30;30(43):2500793. doi: 10.2807/1560-7917.ES.2025.30.43.2500793 (PMC12579318; doi:10.2807/1560-7917.ES.2025.30.43.2500793)
Supplement: Supplementary Material 2 [file 25-00793_SLESAK_Supplement2.pdf]

This supplementary material is hosted by Eurosurveillance as supporting information alongside the article ‘Acute Brugian filariasis in a German tourist after short-term travel to Sri Lanka, 2025’, on behalf of the authors, who remain responsible for the accuracy and appropriateness of the content. The same standards for ethics, copyright, attributions and permissions as for the article apply. Supplements are not edited by *Eurosurveillance* and the journal is not responsible for the maintenance of any links or email addresses provided therein.

<u>407 bp amplicon of *Brugia malayi* from the case depicted in the article</u>

TTTGTCTTTTTTTTTATTTTTATATTTTTATGTTATTTTTGTAAAATGTTTTAGTTTTTTTATCATTTGAAGA
GACAATTAAAATTTGGTTTTTGAACTGGATTAGTACCCAGGTAATCAAAATTAATTAATTCGGGA
GTAAAGTTTTATTTAAACCGAAAAAATATTGACTGACTTTGGATTTTTCTTTGGAATATGCGTATGG
AGAGCCCTCCTTTTTAGTTAACTCTATCGGCACATGTATGATTGTTTAGTTTTTATTTTATTTGTAAT
GCTTTTATGTTTTGTCATTTAAAACAGATATATATTTGGCTTATGGGTTTGTGATCGTGTGTTACTAT
TATTAATTTTTTTTGGATTATTTTTTTATTTTTTTTTGAAATTGGAAAARAAAGTAATTTTTTTTTT
